# Assessment of capillary dropout in the superficial retinal capillary plexus by optical coherence tomography angiography in the early stage of diabetic retinopathy

**DOI:** 10.1186/s12886-018-0778-2

**Published:** 2018-05-08

**Authors:** Ceying Shen, Shu Yan, Min Du, Hong Zhao, Ling Shao, Yibo Hu

**Affiliations:** Department of Zhengzhou Second People Hospital, Ophthalmology, Zhengzhou Eye Hospital, Zhengzhou Ophthalmic Institution, Zhengzhou Hanghai Middle Road No. 90, Zhengzhou, 450000 China

**Keywords:** Optical coherence tomography angiography, Retinal capillary, Capillary dropout, Diabetic retinopathy, Diabetes mellitus

## Abstract

**Background:**

To assess capillary dropout in the superficial retinal capillary plexus (SCP) by optical coherence tomography angiography (OCTA) in the early stage of diabetic retinopathy (DR).

**Methods:**

This study was a cross-sectional observational study. Patients that underwent OCTA examinations in our hospital between November 2015 and May 2016 were included in the study. The subjects were divided into two groups: A) normal controls (41 eyes of 41 subjects) and B) the DR patients (49 eyes of 49 patients with mild non-proliferative DR (NPDR)). The retinal thickness and SCP vessel density were analyzed using built-in software in nine sections of the macular area; whole scan area; fovea; parafovea; and sub-sections of the parafovea, superior-hemi, inferior-hemi, temporal, superior, nasal, and inferior. The correlation between vessel density and retinal thickness was also analyzed.

**Results:**

The SCP density was significantly lower (*P* < 0.05) in mild NPDR patients than in normal controls in all areas, with the exception of the fovea (*P* > 0.05). In the parafovea, superior-hemi, inferior-hemi, temporal, and nasal sectors of group B, the SCP density was negatively correlated with the corresponding retinal thickness (*P* < 0.05). Specifically, as the SCP density decreased, retinal thickness increased.

**Conclusions:**

In the early stage of NPDR, retinal capillary dropout and retinal thickness changes can be clearly captured and analyzed by OCTA. The results confirm a negative correlation between vessel density and retinal thickness in diabetic patients. This noninvasive technique could be applied for DR detection and monitoring. Further study with a larger sample size is warranted.

## Background

Over the last 20 years, time domain and spectral domain optical coherence tomography (SD-OCT) has resulted in the advancement of retinal disease diagnosis [1, 2]. However, SD-OCT is limited in terms of its ability to provide retinal microvasculature information. Fundus fluorescein angiography (FFA) is currently considered the gold standard in retinal vascular network imaging for numerous retinovascular diseases [3, 4]. A novel non-invasive technique, termed optical coherence tomography angiography (OCTA), provides imaging of the retinal vascular network as well as the retina structure, and has been introduced in clinical practice [5].

OCTA, with split spectrum amplitude decorrelation angiography (SSADA), employs motion contrast imaging to obtain high-resolution volumetric blood flow information to generate angiographic images, which does not require the injection of exogenous dyes and provides near-automatic quantification and excellent intra-visit repeatability in the measurement of macular regions [6–8]. In contrast to FFA imaging, which is two-dimensional and explores the retina only on a single plane, OCTA provides a non-invasive approach for three-dimensional (3D) retinal microcirculation imaging [9, 10]. Impressively and notably, the OCTA approach can capture superficial and deep vascular plexuses separately [11].

Vision-threatening retinovascular diseases, such as diabetic retinopathy, retinal vein occlusion, and macular telangiectasia, interfere with retinal microcirculation by modifying the foveal avascular zone (FAZ) size [12].

Therefore, in the present study we took advantage of this non-invasive technique to investigate the superficial retinal capillary plexus (SCP) of the macula in normal and diabetic subjects.

## Methods

This was a cross-sectional observational study. Subjects who visited and received OCTA in the Zhengzhou Second People Hospital clinic between November 2015 and May 2016 were included, that fit within the study criteria. The study was approved by the Institutional Review Board at the Zhengzhou Second People Hospital and carried out in accordance with the tenets of the Declaration of Helsinki. Written informed consent for the study was obtained from all subjects. A total of 90 adult Chinese subjects (90 eyes) were included in the analysis. They were divided into group A (normal controls) and group B with mild non-proliferative diabetic retinopathy (NPDR), diagnosed according to the international definition of DR stages [13]. All subjects underwent comprehensive ophthalmic examination, which included a best-corrected visual acuity (BCVA) test, intraocular pressure (IOP) measurement (CT-80, Topcon Corporation, Tokyo, Japan), slit-lamp biomicroscopy, and pupil dilated fundoscopy. The DR patients underwent FFA in addition. The fundus photographs and FFA were taken by a 55 degree lens (TRC-50DX, Topcon, Tokyo, Japan) at nine fields sites; central posterior, superior, inferior, temporal, nasal, superotemporal, superonasal, inferotemporal, and inferonasal. Then, the manifestations of DR were graded based on the color fundus photos and FFA photos, according to definitions of the DR stages [13]. The inclusion criteria of the study were as follows, 1) normal controls were subjects with 20/20 BCVA or better, with spherical refraction of ≤ ± 6.0 D and cylinder correction of ≤ ± 2.0 D, normal anterior segment and fundus, normal IOP, and no diabetes mellitus; 2) DR patients were subjects that suffered from diabetes mellitus for one to ten years and a stable blood glucose level was maintained (fast blood glucose ≤7 mmol/L and blood glucose after meal ≤11 mmol/L). DR was graded as mild NPDR. The subjects had no previous history of other ocular diseases, surgery, or laser treatment and a BCVA in the 12/20 to 20/20 range. Only one eye of each subject was included in the study.

**Fig. 1:**
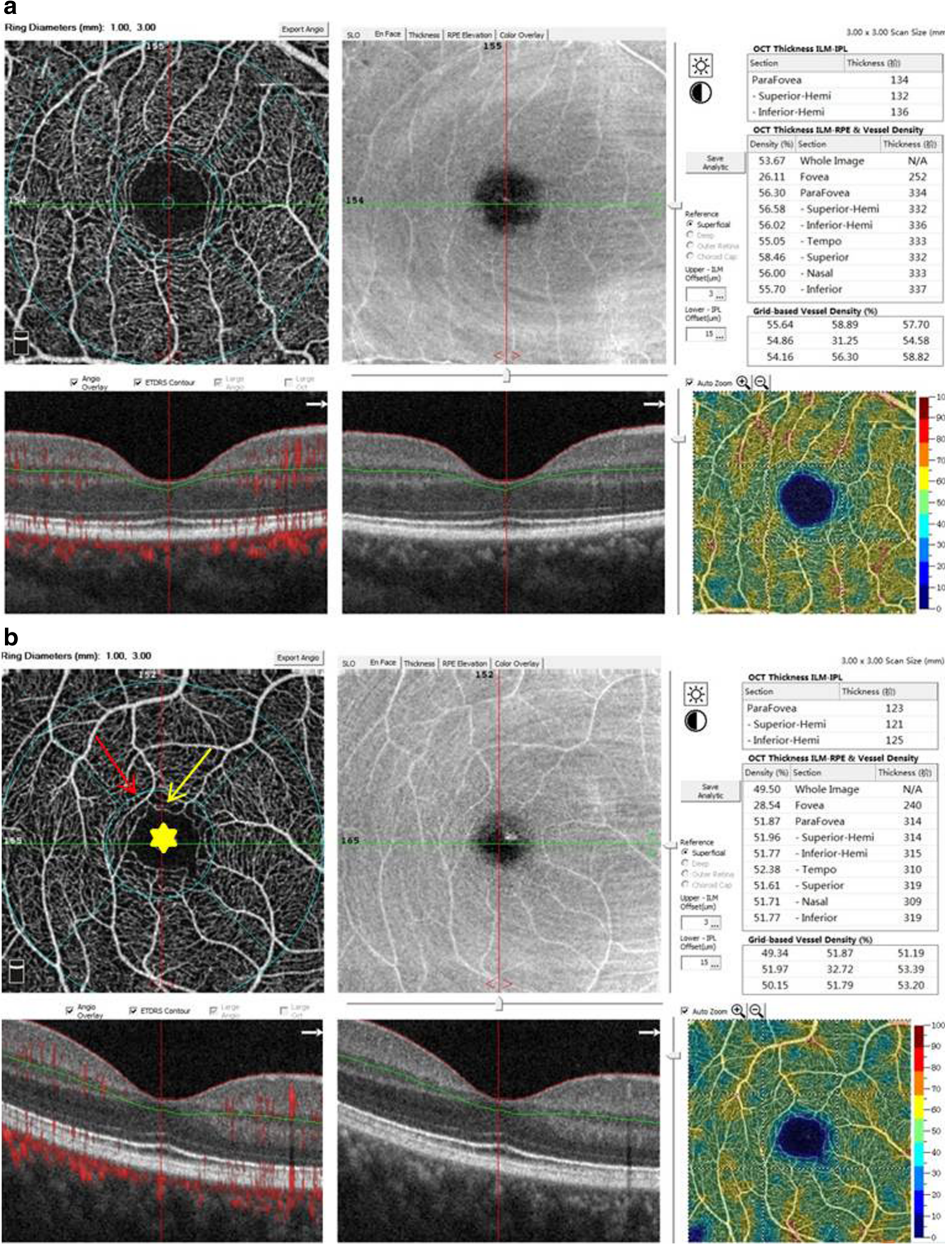
OCTA images of retinal structures and vessel networks, with reports of retinal thickness and vessel density, in a normal subject and a diabetic patient (3 × 3 mm scan area). **a** The structural OCT and angiography of a normal subject did not show abnormalities and the image quality was good. **b** The structural OCT of a diabetic patient did not show abnormalities. Capillary loss (yellow arrow), morphological anomalies (red arrow), and deformed foveal avascular area (yellow star) were demonstrated in an angiography scan

High quality images of the retinal structure and vessel network were obtained by the spectral-domain OCTA, RTVue-XR Angiovue (software version: 2015.1.0.90; Optovue, Inc., Fremont, CA, USA). The built-in software, RTVue-XR Avanti, facilitates automated scan segmentation into the SCP, deep retinal capillary plexuses (DCP), outer retina, and the choroid capillary. Measurements of SCP vessel density on en-face projections were analyzed. The reference plane for the superficial plexus was defined as the inner limiting membrane (ILM) with an offset (from the interface reference) of 3 μm to the inner plexiform layer (IPL) with an offset (from the interface reference) of 15 μm. 3D OCTA scans were acquired over 3 × 3 mm regions. The density occupied by vessels and microvasculature in the selected region were automatically calculated as the percentage of pixels by the built-in software. The software report included the OCT thickness (ILM-IPL and ILM-Retinal Pigment Epthelium (RPE)) and vessel density of the nine sections, i.e., whole image, fovea, parafovea, and sub-sections of the parafovea (superior-hemi, inferior-hemi, temporal, superior, nasal, and inferior; Fig. 1). The software used Early Treatment Diabetic Retinopathy Study (ETDRS) circles to generate the report. Briefly, a small round point at the center represents the fixation point; the diameter of the inner circle is 1 mm and the outer circle is 3 mm; the “whole image” means the whole area of the scan (3 × 3 mm); the “fovea” means the area within the 1 mm circle; and the “parafovea” means the band area from the inner circle to the outer circle; then, the parafovea band is divided into six sub-sections; superior-hemifield, inferior-hemifield, temporal, superior, inferior, and nasal. The foveal avascular zone (FAZ) was used as an anatomic landmark for locating the retinal point of fixation.

### Statistical analysis

Statistical analysis was performed using a statistical software package, SPSS 19.0 (IBM-SPSS Inc. Chicago, IL, USA). Normal distribution of the data was tested. Differences in superficial vessel density between group A and group B were analyzed by the independent t-test. The correlation between the superficial vessel density and the corresponding retinal thickness was studied using Pearson correlation coefficient. The superficial vessel density and the retinal thickness were expressed as the mean ± standard deviation. *P* < 0.05 was considered as statistically significant.

## Results

Group A was comprised of 41 eyes of the 41 normal controls (28 men and 13 women). The mean age of group A was 52.9 ± 8.2 years old (range, 41 to 63 years). Group B was comprised of 49 eyes of 49 subjects (21 men and 28 women) with mild NPDR. The mean age of group B was 56.4 ± 10.1 years (range, 41 to 70 years). There was no statistically significant difference in the mean age between the groups (*P* > 0.05).

### Structural OCT imaging

The structural OCT of the normal and diabetic subjects did not show abnormalities and the image quality was good (Fig. 1a and b).

### OCTA morphology

High quality images, obtained by OCTA, showed clear and organized microvascular networks in normal controls (Fig. 1a). In the diabetic group, at the superficial retinal vascular plexuses level, blood flow alterations, capillary tortuosity and dropout, vasodilation, microaneurysms, vascular remodeling, looser capillary networks with larger and sparser meshes were evident in the macula (Fig. 1b).

### Superficial retinal vessel density and thickness

The 3D nature of OCTA allows for separate visualization of the retina and choroid circulation from the same volumetric scan. In healthy eyes, retinal circulation is located between the ILM and outer plexiform layer (OPL). All participants in groups A and B had adequate OCTA image quality for vessel density analysis. The data were normally distributed (*P* > 0.05).

Compared to group A, the superficial vessel density was lower by 11.61% in the whole image area, 12.90% in the parafovea, 13.13% in the superior-hemi, 12.68% in the inferior-hemi, 11.73% in the temporal, 12.96% in the superior, 13.80% in the nasal, and 13.13% in the inferior, respectively, in the diabetics (Table 1, Fig. 1). All of these differences were statistically significant (*P* < 0.05). There was no statistically significant difference between the vessel density in the fovea section between group A and group B (*P* > 0.05).

**Table 1:** The superficial vessel density and the thickness of ILM-RPE in normal and diabetic subjects ($\overline{x}\pm s$)

Region	group A (*n* = 41)		group B (*n* = 49)	
	Superficial vessel density (%)	Thickness (ILM-RPE) (μm)	Superficial vessel density (%)	Thickness (ILM-RPE) (μm)
Whole image	54.10 ± 2.10	N/A	47.82 ± 4.62^*^	N/A
Fovea	24.48 ± 5.98	233.57 ± 17.90^#^	28.38 ± 5.571^**^	242.35 ± 26.85
Parafovea	56.60 ± 2.19	309.67 ± 12.75	49.30 ± 5.12^*^	313.00 ± 22.27^##^
-Superior-Hemi	56.66 ± 2.25	310.83 ± 13.51	49.22 ± 5.02^*^	313.58 ± 24.46^##^
-Inferior-Hemi	56.54 ± 2.29	308.45 ± 12.65	49.37 ± 5.44^*^	312.28 ± 21.03^##^
-Temporal	55.34 ± 2.24	299.19 ± 14.16	48.85 ± 5.00^*^	305.60 ± 22.68^##^
-Superior	57.70 ± 2.38	315.38 ± 13.82	50.22 ± 5.14^*^	317.65 ± 25.52
-Nasal	56.02 ± 2.50	313.67 ± 13.94^#^	48.29 ± 6.22^*^	314.53 ± 23.45^##^
-Inferior	57.35 ± 2.58	310.55 ± 12.95	49.82 ± 5.66^*^	314.18 ± 20.99

*ILM* inner limiting membrane, *RPE* retinal pigment epithelium

^*^P < 0.05. There was significant differences in vessel density in the parafovea, superior-hemi, inferior-hemi, tempo, superior, nasal, and inferior sections between group A and group B (*t* = 8.593, 8.871, 7.951, 7.767, 8.663, 7.542, 7.941, respectively)

^**^P > 0.05. There was no statistically significant difference between group A and group B (*t* = 0.079)

^#^*P* < 0.05. In group A, the vessel density of the fovea and nasal was positively correlated with the corresponding retinal thickness. Correlation coefficients were 0.689 and 0.312, respectively

^##^*P* < 0.05. In the parafovea, superior-hemi, inferior-hemi, temporal, and nasal sections of group B, the density of the superficial retinal capillary plexus had a negative correlation with the corresponding retinal thickness (correlation coefficient = − 0.358, − 0.359, − 0.322, − 0.374, − 0.358,respectively)

There were no statistically significant differences between the two groups, in terms of retinal thickness, in any section (*P* > 0.05; Table 1, Fig. 1).

### Correlation of vessel density and retinal thickness

In group A, the vessel densities of the fovea and nasal were positively correlated with the corresponding retinal thickness (correlation coefficient = 0.689, *P* < 0.05 and 0.312, *P* < 0.05, respectively). In the parafovea, superior-hemi, inferior-hemi, temporal, and nasal sectors of group B, the SCP density had a negative correlation with the corresponding retinal thickness (correlation coefficient = − 0.358, − 0.359, − 0.322, − 0.374, − 0.358, respectively, *P* < 0.05). Specifically, the lower the vessel density, the thicker the retina. For all other sections, no significant correlation could be found between the vessel density and retinal thickness in either group A or group B (*P* > 0.05).

## Discussion

The results from the present study showed that the image quality obtained from the OCTA device, RTVue-XR Angiovue, was high and was sufficient for analysis using the built-in image analysis software. This technology has improved the visualization of retinal capillaries and micro-angiopathic features, and offers great potential for the study and quantification of retinal microvascular attributes such as vessel density, branching pattern, capillary tortuosity, vasodilation, microaneurysms, and vascular remodeling in both healthy and diabetic subjects [14–16]. The vessel densities of SCP and DCP were lower in diabetic eyes compared with control eyes [17]. There were reports that capillary nonperfusion occurs initially at the level of the DCP [18] and vessel density was more significantly reduced in DCP than in SCP [19]. However, our current device has its limitations and is unable to provide information accurately on the DCP, outer retina, and choroidal vessels due to the projection of the superficial retinal capillary plexus. A similar study reported that measurements of vessel density in the deep retinal layers were influenced by decorrelation tail artifacts within current technologies, and did not appear to have the same diagnostic efficacy as measurements in the superficial retinal layers [20]. Furthermore, the RTVue-XR Angiovue software (Version: 2015.1.0.90) we were using had not been upgraded to perform the analysis of deep retinal vessels and choroidal vessels. That is why the present study only focused on the SCP, where the vessel density could be analyzed accurately. However, a published report demonstrated that a projection-resolved technique detected and quantified the avascular area automatically and accurately [21]. We may apply this technique in our future studies to provide findings in DCP of the subjects at early DR stage.

We explored the correlation of capillary dropout with the corresponding retinal thickness. This analysis relies on the OCTA system providing the structural and functional information simultaneously to accurately locate the abnormalities. The correlation between vessel density and retinal thickness in the macular area of diabetics by OCTA has not been reported in the literature. The report we found was only about the thickness analysis of the retinal nerve fiber layer and ganglion cell layer, and no significant differences were disclosed between diabetics and healthy subjects [19]. In the present study, we found that during the early stage of DR, the density of the superficial retinal capillary plexus reduced significantly in all areas, when compared to normal subjects, with the exception of the fovea. Therefore, retinal capillary dropout is a very important feature observed by OCTA in the early stages of DR. However, in the present study, the retinal thickness did not show a significant difference between normal and diabetic subjects. These findings indicate that compromised circulation in the inner retinal layers during the early stages of DR could be detected before structural retinal changes, for example, edema of the retina. The results from the present study showed that the density of the superficial retinal vessels showed significant intergroup differences in all areas, with the exception of the fovea. The results of the alteration of the FAZ area were inconsistent in some reports. Our result was consistent with the reports in which no significant difference was found in FAZ area of both SCP and DCP comparing diabetic and control groups [18, 19]. However, in another report, FAZ area was greater in diabetic eyes both in the superficial and deep vascular networks [17]. The possible reasons for this interesting finding are that, 1) the fovea is the last area to undergo capillary dropout in the early stages DR, 2) most of the fovea area studied was the FAZ, where there is no vascular network; thus, significant differences could not be found when the capillary dropout was only a small amount in a study with a small sample size, 3) the size of the FAZ was highly variable in the study population [22]. A further study with a larger sample size is warranted.

In the normal controls of the present study, vessel density had a positive correlation with the corresponding retinal thickness in the fovea and nasal sections, with no apparent correlation in others. The finding is consistent with other published work [23]. However, a negative correlation between the vessel density and the corresponding retinal thickness was demonstrated in the parafovea, superior-hemi, inferior-hemi, temporal, and nasal sections of DR patients in the present study. The areas with the least retinal capillaries had the thickest retina, though there was no statistically significant difference in retinal thickness between the controls and the diabetics.

From the results of the present study, combined with the advantages of using OCTA as an imaging technique for retinal vessels and structures, e.g., equivalent to FFA to visualize perifoveal region normal retinal vasculature [24], for patients that have contraindications for FFA and indocyanine green angiography (ICGA), non-invasive, acquires volumetric scans that can be segmented to specific depths, can be obtained within seconds, provides accurate size and localization information, visualizes both the retinal and choroidal vasculature, and shows both structural and blood flow information at the same time, we recommend the application of OCTA for the detection and monitoring of DR in diabetics. Since OCTA has a limited field of view and is unable to view leakage [15], applying OCTA in the early stage of NPDR could be an alternative option to FFA.

The study population in this study was quite small, larger scale investigations are needed to further address the questions that remain unanswered to provide comprehensive guidelines in clinical practice.

## Conclusion

In the early stages of NPDR, retinal capillary dropout and retinal thickness could be clearly captured and analyzed by OCTA. A negative correlation between the vessel density and the corresponding retinal thickness in diabetics was shown in the present study. During the early stages of NPDR, though the retinal thickness was not significantly different with in the healthy control and diabetic groups, the superficial retinal vessel density significantly changed in the diabetic group. This noninvasive technique could be applied by analyzing the retinal vessel density for the detection and monitoring of DR. Further study with a large sample size is warranted.

## Abbreviations

BCVA: Best-corrected visual acuity
DCP: Deep retinal capillary plexuses
DR: Diabetic retinopathy
FAZ: Foveal avascular zone
FFA: Fundus fluorescent angiography
ICGA: Indocyanine green angiography
ILM: Internal limiting membrane
IOP: Intraocular pressure
NPDR: Non-proliferative diabetic retinopathy
OCTA: Optical coherence tomography angiography
OPL: Outer plexiform layer
RPE: Retinal pigment epithelium
SCP: Superficial retinal capillary plexus
SD-OCT: Spectral-domainoptical coherence tomography
SSADA: Split spectrum amplitude decorrelation angiography
